# Odontological Society of Pennsylvania

**Published:** 1896-10

**Authors:** 

**Affiliations:** Odontological Society of Pennsylvania


### ODONTOLOGICAL SOCIETY OF PENNSYLVANIA.

A REGULAR meeting of the Odontological Society of Pennsylvania was held September 12, 1896, at 1415 Walnut Street, Philadelphia, President Peirce in the chair.

### REPORT OF THE DELEGATE TO THE AMERICAN DENTAL ASSOCIATION.

*Dr. Head.*—The American Dental Association was held this year at Saratoga. There were a few good papers, but the chief interest was a step made in the direction of consolidation between the American Dental and Southern Dental Associations. The place of meeting next year will be Old Point Comfort, where the two societies are to hold a joint meeting, and afterwards confer upon the advisability of uniting in one society embracing all the dentists of the United States. As a fitting climax to this decision, a man whom we all respect and honor, Dr. James Truman, was elected president of the Association for the year 1896–1897; and this election was especially fitting, inasmuch as the doctor has advocated such a consolidation for some years past. While his election is a great honor to Philadelphia, every dentist in the country cannot but feel that the American Dental Association did honor to itself in choosing one so eminently fitted to fill the office.

Before reading my paper for this evening, I would like to show you a small pad that has been of great use to me for the last two years.

At one time I used rolls of bibulous paper, then absorbent cotton, and finally they have been combined. The cotton, being enveloped in the paper, is highly absorptive, while the size of the pad can be enlarged or decreased at will. When placed over the mouths of the glands it serves all the purposes of the napkin, without being so bulky.

*Dr. Gaskill.*—Is it any more efficient than cottonoid?

*Dr. Faught.*—The great objection I have found to cottonoid has been the shortness of its fibre. It is exceedingly unstable, and the peculiar linty dust annoys the patient's throat, especially if they are at all sensitive. I gave up the use of the cottonoid and have adopted a method similar to Dr. Head's.

*Dr. Gaskill.*—My patients have never been troubled by dust or lint when I have used it.

*Dr. Broomell.*—My experience has been somewhat similar to that of Dr. Faught. Further than its disturbing effect on the patient's throat, the cottonoid has a tendency to mix in with the filling, especially gold. I found it also objectionable on account of its clinging to the instrument.

Dr. Head then read his paper on "The Application of Crown-Work to the Manufacture of Metal Plates."

(For Dr. Head's paper, see page 637.)

DISCUSSION.

*Dr. Head.*—I am sorry Dr. Bonwill is not here to-night, for the specimens show that I have used his method of putting on spuds, which, extending over the teeth, prevent mastication from injuring the gum and cutting the enamel, the plate being held absolutely in position.

*Dr. Truman.*—Is the thin backing gold plate?

*Dr. Head.*—Twenty-two carat gold, because it is a little less likely to burn.

*President Peirce.*—I do not understand why Dr. Head speaks of these as Dr. Bonwill's spuds. They were used by Dr. J. D. White before Dr. Bonwill came into practice. They were on the first case that I ever saw when I was a student.[1]

*Dr. Head.*—The first plate that I ever saw that had them was made by Dr. Bonwill, and they seemed so valuable that I never omitted an opportunity to use them where possible.

*President Peirce.*—When I was a student a case came into my hands with three clasps on it, and each clasp had a spud.

*Dr. Head.*—They make all the difference between cutting and non-cutting of the teeth.

*Dr. Warren.*—I want to indorse the method of Dr. Head's. I have used it for possibly two years. My brother and I performed the same operation in our laboratory, with the exception that we used pure gold thirty-four thickness, and one plate. It should be brought over the edges. That is the weak point. Bunch it down with a sharp chisel and make a slight spur. That spur will hold the backing in place, which seems preferable to bending the pins.

*Dr. Head.*—It is a question of having a thick backing. Thirty-four is hardly sufficient. I prefer to have a reinforced backing on my work, at least at the bottom, as it can then be ground to any shape desired.

*Dr. Broomell.*—Dr. Head advocates bending the pins. I think

---

[1] Dr. Peirce in his remarks on Dr. Head's paper informed the Society that I was not the first to place *spuds* upon metal plates. I never said I was. Dr. J. D. White, I know, did it long before. What I do claim is, there is no evidence that any one ever made clasped plates on the plan I devised and gave to the dental world and published in the proceedings of this Society in this journal six or more years ago, and I made the spud of absolute importance in every case of these removable sectional bridges. But the spud is of no use unless one knows how to solder a clasp to the plate and all the conditions required in my description of such work. I claimed then that, while I used an old thrown-away spud, the combination was entirely new, and the laws of invention recognize my right, although it was never patented.

W. G. A. Bonwill.

Philadelphia, September 21, 1896.

he said you may even rivet them. In my mind, bending is dangerous, particularly if a pair of pliers be taken and a pressure exerted that is continually pulling from the porcelain. When you apply the heat the platinum pin will make that pressure stronger, and checking of the tooth is apt to occur. Dr. Warren's plan, I think, is an excellent one. The backing should be held lightly against the tooth, and no strain should be exerted on the porcelain.

*Dr. Head.*—The question of bending the pins is not one that I insist upon. As regards the advisability of riveting pins, it hardly, in my opinion, needs any defence. Dr. Warren's process is excellent where the backing fits the tooth, but it does not seem to be a method applicable where there is difficulty in making the backing hug. The advantage in riveting the pins lies in the fact that the backing can be made slightly concave and then brought down accurately against the tooth, which is riveted on soft lead. Dr. Broomell says bending the pins down on the backing is dangerous, as the expansion of the platinum in heating would be likely to cause checking. With this view I cannot agree. As the expansion of the pin would be much greater along its length than in its diameter, the tendency in heating would be to push it away from the porcelain, and the danger from subsequent contraction would be no greater than in any other method of treating the pins. The only risk in bending the pins lies in the possibility of fracturing the porcelain, and if there is sufficient tooth body there is no reason, in my opinion, why this method should not be employed.

*Dr. Truman.*—For twenty years I riveted the teeth before soldering, and I never had any trouble. It is simply a matter of use, not to rivet too tightly, and I abandoned it because I thought the solder sometimes failed to penetrate behind the pin. Then I began splitting the pin. I do not know that it is any better. It seemed to be accompanied with less difficulty. I do not quite understand Dr. Head's process of working the thin plate first to make it completely fit down on a tooth that is ground to the plate. I always had difficulty in running solder into a place of that kind, and in placing pieces of metal to fill a space was never satisfactory. The double plate is a very old affair. I remember it as far back as 1853, when Dr. Reynolds introduced the double plate in Philadelphia (the most beautiful work ever seen in this country up to that period), and the large proportion of sets I made, when these were confined to gold or silver, were made in that way, but every tooth had to be separately prepared, soldered, finished up, and placed back again. A great deal of work, but a beautiful and strong case. I have work in mouths now that were fitted in that way.

*Dr. Head.*—I use the double backing, but do all the soldering at one heating.

*Dr. Gaskill.*—My method in backing the tooth follows Dr. Warren's plan, but I make three spurs. Since using that process I have had much less trouble with the teeth cracking during the soldering process.

*Dr. Boice.*—I think that Dr. Truman struck the nail on the head when he said that riveting prevented the solder from flowing around the pin. The object of the spur is merely to hold the backing in place. When I was working in the laboratory I was instructed to rivet them, but it did not satisfy me. While experimenting one day, I made spurs, and I have been using them ever since. It is easier to put the spurs on the side. I hardly think the riveting will break the tooth. It is the injudicious heating that causes this effect. Dr. Head said that one disadvantage of attaching teeth to gold plates with rubber lay in the fact that space was formed by shrinkage of the rubber. If one properly adapts a piece of rubber with sufficient pressure to a clean tube or plate, and vulcanizes it, I defy any gentleman to pull off that rubber, and if there is a leakage it is the fault of the materials or operator. If sufficient pressure is used in hardening, the rubber runs away from everything. Its tendency is to assume a globular form. There are so many people finding fault with rubber that I should like to bear my testimony that it is a most valuable material in skilful hands, and too often the accusations brought against it should be laid at the door of unskilful mechanics.

*Dr. Head.*—I would speak still on the subject of riveting teeth, although I seldom use the process. I still bend them at times, although I prefer the splitting. I am at a loss to see how the riveting of the pin can in any way prevent the solder from flowing around it. The solder will enter into the substance of the plate. If the pin and plate have been well polished, and the whole boraxed and the heat properly applied, I cannot understand why the solder should not flow. I have had no difficulty in this direction.

*President Peirce.*—The question of the solder not flowing under the rivets is usually due to the whole body not being sufficiently heated. When I worked in the laboratory I sometimes burned the teeth. Frequently, when soldering, I placed my whole case in my muffle. The investment and teeth were brought up to such a degree of heat that the solder would freely flow over the rivets and the union was perfect.

*Dr. Truman.*—While I was absent this summer I met a jeweller in Massachusetts, a wholesale manufacturer. I inquired of him how they managed the soldering with large numbers of workmen, and economically as far as power was concerned, and how they controlled the air-blast. He said the old method of mouth blow-pipe had practically gone out of use, they preferring the power blower. This costs from eighteen to twenty dollars, the air being forced into an ordinary tin pipe around the tables, and from this attachments were made to the blow-pipe, adapted for both gas and air. He said, as soon as the pipe was full of air the excess would be forced back into the blower, so that there was never any danger of the tin pipe bursting. I have been looking for years for some method by which students could use conveniently compressed air. I had thought of large tanks of some kind. But this is a simple process and can be used in colleges very economically, not requiring great power.

*Dr. Warren.*—While I was attending the British Dental Society, this summer, Dr. Tomes told me of the case of a man who had been experimenting with the Röntgen discovery, the so-called X-rays, on the right cheek of his assistant. There were five or six sittings of five minutes each. To his astonishment he found that the patient lost his beard entirely on the side of the face touched by the rays, and that the life of the hair was destroyed. As it is an entirely painless operation, it may take the place of the electric needle now used for that purpose, which is not an altogether satisfactory instrument.

Dr. Boice then introduced the following resolutions concerning an election irregularity that occurred at the July meeting of the Pennsylvania State Dental Association:

WHEREAS, At the last meeting of the Pennsylvania State Dental Society, held at Bellefonte, Pa., on the 7th, 8th, and 9th of July, 1896, a crime was committed which, if allowed to pass unnoticed, would place a lasting blot upon the name of the profession in Pennsylvania; and

WHEREAS, All members of the Pennsylvania State Dental Society must feel the disgrace and hold in utter horror those who caused it; therefore be it

*Resolved*, That we, as members of the Odontological Society of Pennsylvania, hold it as our opinion that those who are responsible for this disgrace to us, as dentists and citizens of the commonwealth of Pennsylvania, should be punished.

These were seconded by Dr. Head, and elicited the following discussion:

*President Peirce.*—You have the resolution as read by Dr. Boice. What is your pleasure?

*Dr. Gaskill.*—Mr. President, I was not present at this meeting, but this seems a great step for this association to take without much consideration. I do not know that it would be advisable for this Society to go on record as interfering with the State society.

*Dr. Boice.*—Mr. President, I introduced the resolution. It is in the province of any one, it is the duty of any man in the State, to argue and to express his opinion. Nobody denies that it is a crime, and it is our place to put ourselves on record as being absolutely opposed to such proceedings. Is there any man who for a moment believes that a crime was not committed?

*Dr. Truman.*—I was not present, but I have heard much in regard to it. It seems to me that a resolution of that sort should be worded with some caution. The crime should be specifically stated.

*Dr. Head.*—It is undenied and well known that there was a discrepancy in the report handed in to the Association, and all dentists who hold the honor of dentistry, as well as all who hold the honor of their country dear, inasmuch as the State laws were broken, should consider it a personal matter that whosoever was guilty should be at least condemned by the united voice of their fellow-members.

*Dr. Truman.*—I do not want it to be understood that I desire to condone this matter. I think it was one of the worst complications that ever happened in this or in any other State. In my view, this association has the proper right to criticise it severely. The point is, what is the best course to pursue?

*Dr. Gaskill.*—That was the reason for the remarks I made. I agree with Dr. Boice and Dr. Head. We cannot afford to have an act like that pass unnoticed.

*Dr. Head.*—In regard to the criticism made on the use of the term "crime," my reply would be that there is, in my judgment, no other word for it. It would seem useless to use a milder word. It is hardly our province here to-night to take notice of who did it. It is simply our duty to state plainly and positively that we are in no way a party to it. And, inasmuch as the members of the State Association did not openly speak their minds as an organization against it, they have left themselves, in my opinion, open to suspicion. I am very much opposed to allowing this Society to remain silent on this vital issue.

*President Peirce.*—I think no one who is familiar with the transaction of the meeting can for one moment have any apology for it, but there are several facts that must be recognized. In the first place, more than half the members had left the hall and gone home before the fact was recognized. It was the last act of the society. The subject was before the council and the council had not yet made their report to the society. Now it is a question that must come up before the State society next year, and, while there is no impropriety in this Society's expressing its abhorrence of the transaction, beyond that, it seems to me, we cannot go, because we are forestalling the duty that belongs to the State Association.

At Dr. Warren's suggestion the words "in our opinion" and "if allowed to pass unnoticed" were inserted, and the resolutions as amended were unanimously carried.

Adjourned.

JOSEPH HEAD,
*Editor Odontological Society of Pennsylvania.*

